# Evaluating the relationship between binge drinking rates and a replicable measure of U.S. state alcohol policy environments

**DOI:** 10.1371/journal.pone.0218718

**Published:** 2019-06-25

**Authors:** Diana Silver, James Macinko, Margaret Giorgio, Jin Yung Bae

**Affiliations:** 1 Department of Public Health Policy and Management, College of Global Public Health, New York University, NY, NY, United States of America; 2 Departments of Health Policy and Management and Community Health Sciences, Fielding School of Public Health, University of California Los Angeles, Los Angeles, CA, United States of America; 3 Steinhardt School of Culture, Education and Human Development, New York University, NY, NY, United States of America; Florida State University College of Medicine, UNITED STATES

## Abstract

Excessive alcohol consumption contributes significantly to premature mortality, injuries and morbidity, and a range of U.S. state policies have been shown to reduce these behaviors. Monitoring state alcohol policy environments is essential, but methodologically challenging given that new laws may be passed (or repealed) each year, resulting in considerable variation across states. Existing measures have not been made public or have only a single year available. We develop a new replicable measure, the state alcohol policy score, for each state and year 2004–2009, that captures the essential features of a state’s evidence-based alcohol policies. We evaluate its similarity to two existing alcohol policy measures and validate it by replicating findings from a previous study that used one of those measures to assess its relationship with several binge drinking outcomes. Estimates of the association between one-year lagged state alcohol policy scores and state binge drinking outcomes, obtained from the 2005–2010 Behavioral Risk Factor Surveillance System surveys (n = 440,951, 2010), were produced using Generalized Linear Models that controlled for state and individual-level co-variates, with fixed effects for year and region. We find a 10-percentage point increase in the state alcohol policy score was associated with a 9% lower odds of binge drinking (aOR = 0.91, 95% CI 0.89, 0.92; N = 1,992,086), a result consistent for men, women and for most age and race subgroups. We find that gender gaps in binge drinking behaviors narrowed in states with higher state alcohol policy scores. These results were nearly identical to those found in other studies using different scores obtained with the aid of expert opinions. We conclude that the score developed here is a valid measure that can be readily updated for monitoring and evaluating the variation and impact of state alcohol policies and make available our state scores for the years of the study.

## Introduction

Excessive alcohol consumption—which can include heavy drinking, binge drinking, repeated binge drinking, and the quantity of drinks consumed in a single binge episode—contributes significantly to premature mortality, injuries and morbidity in the United States [1]. While some federal efforts have been made to regulate alcohol consumption, U.S. states are largely responsible for establishing policies to reduce alcohol harms. However, policies such as restricting prices and sales, taxation and provision of alcohol, and penalties for driving while intoxicated vary widely across states [2]. There is a strong body of evidence for the effectiveness of many of these policies, but far less evidence regarding their cumulative impact, or whether gaps in policy adoption across states are meaningful [3, 4]. Monitoring the effectiveness of state policy environments is essential to meeting the Healthy People 2020 goals of a 10% reduction in average alcohol consumption and a 24% reduction in binge drinking by adults [5]. Ongoing policy surveillance is especially important given that evidence for some alcohol control policies is more than a generation old and changes in the demographic, economic and social context in some states—as well as social changes in alcohol consumption—may have altered their effectiveness.

Efforts to monitor alcohol policy environments across states face methodological challenges given the number of different policies and the varied features of the policies that states adopt. Scholars have created policy indices to try to capture the influence of the overall alcohol regulatory environment since the effectiveness of any new policy is likely to be influenced by the existing combination of policies directed at controlling different aspects of alcohol availability or price for example [6]. This approach weighs each policy equally even though some policies may be more effective than others or contain different features that may alter their overall effectiveness. Erickson et al [7] assessed state alcohol policies in 18 different domains and developed a coding scheme to assess each one’s enforceability and population reach. These measures were then summed for each state to calculate scores for each state reflecting the restrictiveness of its overall alcohol policy environment. However, the article did not test the association of this score with state-level alcohol consumption or problem drinking behaviors. In another approach Naimi et al. convened alcohol policy experts to identify relevant alcohol policies to create efficacy and implementation ratings for each policy using a modified Delphi method [8]. The team used these ratings for each law as weights in calculating composite alcohol policy scores (APS) for each state and year [8]. Then, the team analyzed the association between these scores and state-level binge drinking prevalence over the period 2005–2009 and found that a 10-percentage point increase in the state level scores was associated with a 10% lesser odds of binge drinking [9]. The estimate was generally consistent across gender subgroups, and between whites and non-Hispanic other subgroups, but not between white and non-Hispanic Blacks or Hispanics [9].

These studies and Naimi et al.’s research in particular, advanced understanding of the role the entire sum of alcohol policies can play in reducing excessive alcohol consumption. However, replicating this work is difficult because there are no public use files containing the policy ratings for each state. Updating the score or replicating it to include new policies and time periods would be laborious, expensive and impractical, given the difficulties in convening and re-convening an expert panel.

Consistent with the call to replicate findings and make data available in an open science framework, we develop a state alcohol policy score 2004–2009 that relies on public use data supplemented with original legal research and employs a transparent coding approach that captures the complexity of each law, similar to Erickson [7]. Instead of relying on expert weights as in previous studies, we assign points to specific features of each law (available by reviewing the legal text) that could add or detract from its overall effectiveness. We then sum the points to create a composite score for each year and state for the period 2004 to 2009. We assess the validity of this score by comparing it to state scores derived by Naimi and Erickson for selected years. We then use our score to replicate Naimi et al’s findings regarding the relationship between state alcohol policy environments and drinking behaviors. We conclude with a discussion of the role of state policies and their measurement, as well as consideration of other state and individual level factors that remain important predictors of excessive alcohol consumption. Accompanying this article are the state-year scores and coding scheme (S1 Table) that can be used to replicate and extend our findings.

## Data and methods

We constructed a State Alcohol Policy Score (SAPS) for every state and year from 2004 to 2009. Hewing as closely as possible to the policies that appear in Naimi et al’s previous work [8], we retrieved public use data and used original legal research to capture alcohol policies and legal provisions (often different provisions contained within the same law) intended to regulate alcohol price, availability, and consumption. Policies present in both approaches include those recommended by the U.S Community Preventive Services Task Force or which have strong empirical evidence of reducing excessive drinking behavior or alcohol-related harms [10–15]. Our coding approach is informed by a thorough reading of the state statutes by the attorney on our team.

The final score was composed of 18 categories within 4 main domains: Alcohol pricing (alcohol control systems, wholesale pricing practices, retail price restrictions, total beer tax); Sales and retailer restrictions (Sunday sales ban, keg registration laws, hours during which alcohol cannot be sold, beverage service training laws, dram shop laws, pregnancy and alcohol warning signs); Driving-related laws (DUI penalties, open container laws, ignition interlock installation requirements, graduated driver’s license laws); and underage alcohol furnishing and possession laws (underage purchasing, underage alcohol possession laws, false ID penalties, and laws prohibiting the furnishing of alcohol to minors). Unlike Naimi et al, we do not include the ratio of alcohol retailers to population, since the ratio does not capture a specific policy tool. We note that other scholars and agencies have specified a smaller set of the policies with a strong evidence base [15, 16] but we include this larger set of policies to replicate Naimi et al’s approach as closely as possible. For a detailed list and coding protocol, see S1 Table.

Data comprising 12 of the 18 categories used to construct the score were obtained from the Alcohol Policy Information System (APIS) [17]. Graduated drivers’ license laws came from the IIHS [18]. For laws in the five additional categories, a public health attorney conducted original legal research using the online legal databases Westlaw and Hein Online. Based on citations for prior versions of the statutes available in the Westlaw historical notes, the full texts of the laws during the period of 2004–2009 were retrieved. The attorney reviewed the texts and coded each law’s specific provisions, using previously established protocols [19]. Each of the 18 categories was assigned a maximum number of points based on the presence of specific provisions contained within the legal texts. Our coding scheme (see S1 Table) uses points to capture the application of the law to different types of alcohol sold (beer, wine, spirits), the stringency of the law (the presence of exemptions), the type of legal penalty for violation (civil, criminal) and legal standard (e.g., knowledge of the law).

To operationalize the resulting score, we calculated the percentage of components that states had in place in a given year for each legal domain, where a higher percentage represented greater (or more comprehensive) regulation. We then calculated the mean score for the 18 alcohol-related legal categories for each state for each year. The overall score had a possible range of 0% (no laws in place) to 100% (all laws with all provisions in place). For multivariable analyses, the score was divided by 10 to facilitate interpretation.

To examine the SAPS’ ability to predict state-level drinking behaviors, we test their association with three high-risk drinking outcomes. Prior to 2006, binge drinking was defined as 5 alcoholic drinks in one sitting for both men and women. As of 2006, binge drinking has been defined as drinking 4 or more alcoholic drinks in a sitting for women and 5 or more for men on at least one occasion during the previous 30 days [20]. Recent frequent binge drinking is defined as engaging in 5 or more episodes of binge drinking in the previous 30 days. High volume binge drinking is defined as consuming 10 or more drinks in at least 1 binge drinking episode in the previous 30 days. We assess these three main high-risk drinking outcomes using self-reported data from the 2005–2010 Behavioral Risk Factor Surveillance System (BRFSS)—a telephone-based survey of U.S. adults aged 18 and older designed to be representative of all 50 states [21]. Data for this study were assembled, coded and analyzed March-October 2018.

### Analytic approach

First, we present descriptive statistics for the SAPS score by year for all 50 states and compare the distribution of these scores across states in 2008 and 2009 to the alcohol policy state scores developed by the two teams described above, Naimi et al and Erickson et al (only 2009 data were available) [7]. We use graphical methods and parametric and nonparametric statistical tests to assess the similarity between the three sets of scores.

Next, we fit a series of multivariable models with individual and state-level covariates. Individual-level covariates were obtained from the 2005–2010 BRFSS [21], and included categorical variables for age, gender, race/ethnicity, educational attainment, employment status, marital status, and household income level. State-level population estimates for the percent female, percent non-white, percent aged 21 or older, median household income, population density, and state total population were obtained for each state-year from the American Community Survey (http://www.census.gov/acs/). We included the number of police per 1,000 capita for each state-year, obtained from the FBI’s Uniform Crime Reporting database (https://www.fbi.gov/services/cjis/ucr).

We use survey-adjusted multivariable binomial generalized linear models (GLM) with robust variance estimation to calculate odds ratios (ORs) and 95% confidence intervals (CIs) for the relationship between each high-risk drinking outcome and the SAPS for the entire BRFSS sample.

To establish temporality between the state alcohol policy score and behaviors, we lag the score by one year, thus covering the period 2005–2010. We examine the relationships between the SAPS and the three study outcomes overall and among the gender, age, and race/ethnicity categories. All models include BRFSS survey weights (divided by the number of years in the sample) to ensure state representativeness. All multivariable models control for all individual-level covariates identified above (with the exception of instances where the analysis was stratified on one of these factors) for all state-level covariates, and include fixed effects for each year to capture secular trends that might affect all states, and fixed effects for the four U.S. Census geographic regions of the country to control for regional behavioral patterns and other unmeasured time invariant factors. Analyses were conducted using Stata 15 statistical software [22].

### Results

The mean SAPS grew from 38.7 in 2004 to 41.7 in 2009, increasing slightly after each year of observation, and the 3-point difference in the mean score 2004–2009 was statistically significant (p<0.001) indicating that alcohol policy environments across states grew stronger on average over the period (see Table 1). Still, in each year, scores varied widely across states, with scores ranging from a low of 17.2 (WY, in 2004) to 64.2 (UT, in 2009).

**Table 1:** Mean State Alcohol Policy Score (SAPS) by year 2004–2009.

Year	State Alcohol Policy Score		
	Mean (SD)	Min	Max
2004	38.7 (8.2)	17.2	58.5
2005	39.1 (8.1)	17.2	59.3
2006	40.3 (7.4)	19.5	58.4
2007	41.2 (7.6)	19.5	60.5
2008	41.6 (7.6)	19.4	60.4
2009	41.7 (7.8)	19.4	64.2
Difference 2009–2004	**3 (.47)** ***		

*** p<0.001 from t-test

**Fig 1:**
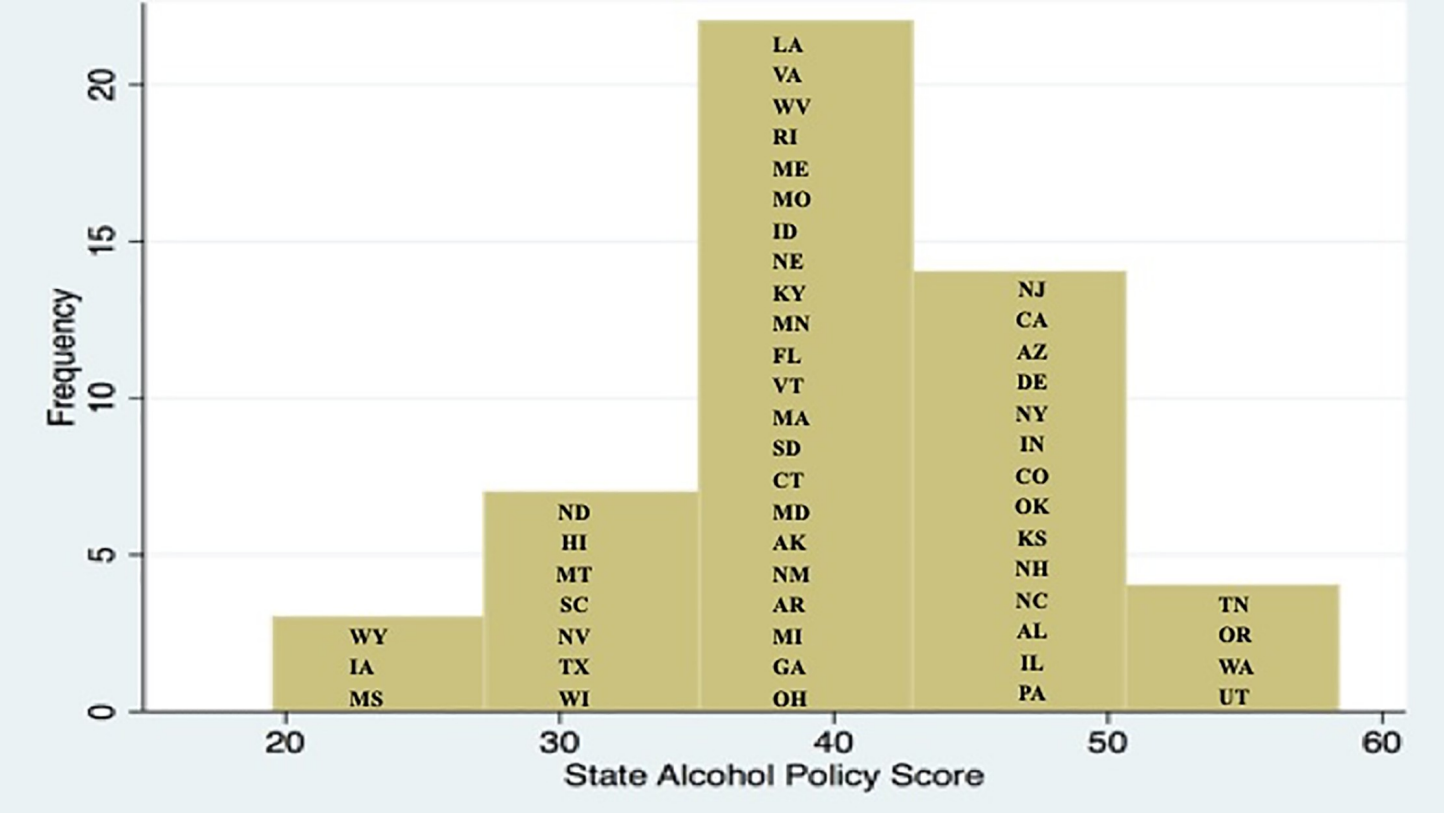
Distribution of State Alcohol Policy Score (SAPS), 2007.

Fig 1 displays the SAPS for one year (2007), which resembles a normal distribution with scores ranging from 19.5 to 60.5. In this year, the three states with the lowest scores were IA, MS, WY. There were also a small number of outliers on the high end, representing states with the strongest policy environments (TN, OR WA, UT). The types of laws adopted varied as well: within the overall score, the two policy domains with the highest means were driving (58.49) and underage (47.88), while alcohol pricing had the lowest mean score (24.79) (see S1 Table).

Table 2 shows that the means, minimum and maximum values for the SAPS, Naimi’s and Erickson’s scores. The SAPS and Naimi scores do not differ significantly in terms of these values. Given the smaller set of policies that score includes, the Erickson mean score is lower than the others as expected, with a smaller difference between the min and max values.

**Table 2:** Descriptive means and distribution of SAPS, the APS (Naimi et al 2014), 2008 and 2009 and Erickson et al’s scores (2009).

	2008		2009	
	Mean (sd)	Min, max	Mean (sd)	Min, max
SAPS	41.59 (7.63)	19.45, 60.41	41.69(7.81)	26.49, 65.86
Naimi et al	42.85 (8.56)	19.38, 64.15	43.11 (8.55)	26.37, 66.76
Erickson et al			34.74 (4.80)	28.0, 51.0

Table 3 presents the correlation coefficients for all three scores. Pearson correlation coefficients were all statistically significant at the (p<0.001) level. Nonparametric tests (Spearman rank-order correlation and Kendall’s Tau) also reject the hypotheses that each pair of values is independent. (p<0.001). A visual comparison of our SAPS score with the Naimi and Erickson 2009 scores appears in S1 Fig [8]. S2 Table compares each score by separating each state into quintiles based on their 2009 score. The SAPS and Erickson scores did not differ by more than one quintile for any state, but the APS scores for two states differed by 3 quintiles and differed by 2 quintiles for another ten states, when compared with the SAPS.

**Table 3:** Correlation coefficients for the SAPS, Naimi et al and Erickson et al scores, 2009.

	Kendall’s Tau	Spearman	Pearson
Naimi et al v SAPS	0.4678	0.6638	0.6621
Erickson et al v SAPS	0.3633	0.5317	0.5907
Naimi et al v Erickson et al	0.3976	0.5712	0.6146

Note: all coefficients statistically significant at the p<0.001 level.

Table 4 shows results from multivariable models predicting three different high-risk drinking behaviors. In the full model for the entire population 2005–2010, the SAPS was significantly associated with a reduction in all three high-risk drinking outcomes: a 10 percentage point increase in the SAPS was associated with a 9% lower odds of binge drinking (aOR = 0.91, 95% CdsI 0.89, 0.92), a 12% lower odds of high frequency binge drinking (aOR = 0.88, 95% CI 0.86, 0.91), and a 15% lower odds of high volume binge drinking (aOR = 0.85, 95% CI 0.83, 0.88). Estimates for each covariate in the full model are shown in S.3 Table.

**Table 4:** Adjusted odds ratios of high-risk drinking with a 10-percentage point increase in 1-year lagged SAPS, 2005–2010.

	Descriptive statistics		Any binge drinking, past 30 days		5+ binge drinking episodes, past 30 days		10+ drinks in a single episode, past 30 days	
	%^1^	N, 2010^2^	aOR	95% CI	aOR	95% CI	aOR	95% CI
Overall								
(full model)	100	440,951	0.91	0.89, 0.92	0.88	0.86, 0.91	0.85	0.83, 0.88
Gender								
Female	51.36	274,598	0.91	0.89, 0.93	0.89	0.84, 0.94	0.85	0.79, 0.91
Male	48.64	166,353	0.90	0.89, 0.92	0.88	0.85, 0.91	0.85	0.82, 0.88
Age								
18–20	5.93	9,512	0.92	0.84, 1.00	0.83	0.71, 0.97	0.86	0.75, 0.99
21–34	24.35	40,673	0.89	0.86, 0.91	0.86	0.82, 0.91	0.83	0.79, 0.87
35–64	52.80	241,895	0.92	0.91, 0.94	0.90	0.87, 0.93	0.88	0.84. 0.91
65+	16.93	148,871	0.86	0.83, 0.90	0.85	0.78, 0.92	0.78	0.69, 0.90
Race/ethnicity								
NH White	65.97	349,606	0.90	0.89, 0.91	0.88	0.85, 0.91	0.86	0.83, 0.89
NH Black	9.78	33,429	1.00	0.94, 1.07	0.99	0.87, 1.13	1.03	0.90, 1.20
Hispanic	13.81	28,025	0.92	0.86, 0.99	0.81	0.70, 0.95	0.80	0.70, 0.91
NH Others	6.84	23,753	0.92	0.86, 0.99	0.80	0.70, 0.93	0.80	0.70, 0.91

Data from BRFSS. Numbers are adjusted odds ratios and 95% confidence intervals from stratified multivariable GLM models that each control for individual-level factors (sex, educational attainment, age, race/ethnicity, marital status, household income, employment status) state-level factors (percent poverty, police per 1000 persons, population that is white, percent of population 21 and over, percent female, population density, geographic region), and year, adjustment for survey design, and include individual weights.

^1^Average proportion over the entire period

^2^Unweighted number, 2010 only. Total unweighted sample size for full model = 1,992,086.

**Fig 2:**
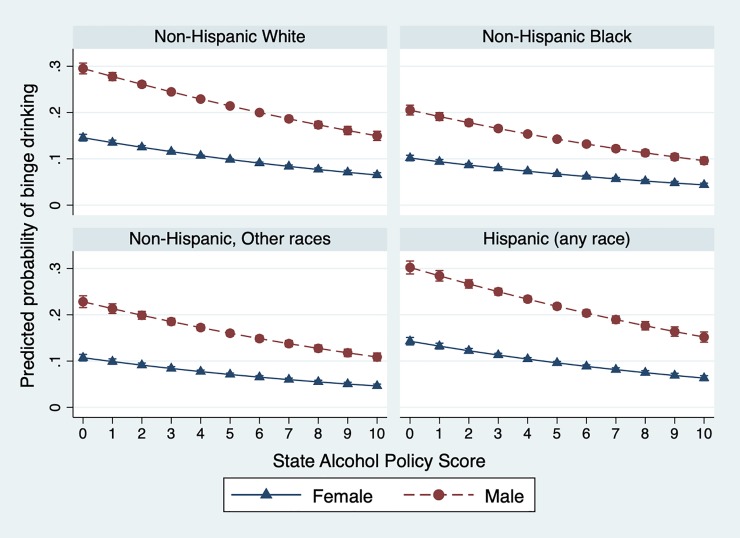
Predicted probability of binge drinking, by SAPS score, race/ethnicity, and gender, 2005–2010. Data from BRFSS and other sources. Predicted probability from full GLM regression model controlling for individual-level factors (sex, educational attainment, age, race/ethnicity, marital status, household income, employment status) state-level factors (percent poverty, police per 1000 persons, population that is white, percent of population 21 and over, percent female, population density, geographic region), and year, adjustment for survey design, and including individual weights.

Fig 2 presents predicted probabilities from the full model. For each race/ethnicity category, lower SAPS scores are associated with higher probability of binge drinking. This probability declines as the SAPS increases in strength. The probability of any binge drinking is higher for males in each group, but there are important sub-group differences in the highest and lowest rates as well as the gap between men and women. An increase in the state SAPS from 0 to 10 would be associated with a 30% to 18% reduction in binge drinking among the group with the highest probability of that behavior (white, non-Hispanic males). We note that the average change in SAPS observed over this period was only 3 points out of 100, but there is a 45- point gap between the state with the lowest and highest SAPS in 2010.

In subgroup analyses (Table 4), we found little difference by gender for any of the high-risk drinking outcomes. However, we found that the magnitude of the relationship between policy environments and adult binge drinking increased for older age groups: the relationship between the SAPS and binge drinking was greatest among individuals aged 65 and older (aOR = 0.86, 95% CI 0.83, 0.90) as compared to other age groups 21+ for all three outcomes. The inverse relationship between state alcohol policy scores and the three binge drinking outcomes was statistically significant for whites, Hispanics, and for those in the “other race” group. In contrast, none of the point estimates for the three high-risk drinking outcomes were statistically significant among African-Americans.

## Discussion

Using an alternative method of measuring the state alcohol policy environment, these findings confirm that states vary considerably in the number and content of their policies to reduce excessive alcohol consumption and the negative outcomes associated with it. On average, over the period 2004–2009, states adopted more or stricter policies, and gaps between states adopted narrowed slightly over time between leaders and laggards. Still, these gaps remain large and persistent.

We find that the strength of a state’s alcohol policy environment, as measured by the SAPS, is associated with reductions in binge drinking outcomes for the state population overall, for men and women separately, and for nearly every age and race subgroup. For each 10-percentage point increase in a state’s SAPS, the odds of any binge drinking fell by 9%, with stronger effects for other binge drinking outcomes. Men and women shared almost identical gains for all outcomes, as did Non-Hispanic Whites, Hispanics and Non-Hispanic others. Reductions in binge drinking were also large for those over age 21, for frequent binge drinking and 10 or more drinks in a single episode, and statistically significant for those under 21.

Multivariable models estimating the impact of the SAPS on binge drinking rates produce remarkably similar results from models that use the APS [9]. Significant findings between these two studies differ only for outcomes for those 18–20 years of age, and for those who identify as Hispanic, where we find patterns consistent with white respondents. These differences may be related to investigating relatively rare outcomes in the small sample size for these groups (see the confidence intervals for these groups in both papers), such that the findings are significant at the p-value of 0.10. In addition, we did not find a relationship between alcohol policy environment and binge drinking for non-Hispanic Blacks. Rates of binge drinking are lower among this group, which could partially explain this finding, but this is an area for further investigation. Most importantly, the results presented here confirm the principal findings of studies conducted using the score developed by Naimi et al: between 2005–2010 states with stronger alcohol policy environments experienced reductions in any binge drinking in the population overall and in nearly every population subgroup.

Our successful effort to validate our score by replicating previous findings provides strong evidence that our alternative method is viable. Our coding captures some of the nuance of the constructs of Naimi et al’s efficacy and implementation ratings, but allows for independent teams to build scores to investigate different time periods, incorporate new knowledge regarding existing laws, and add additional laws through a consistent coding schema. An advantage of our method is that it uses data that any research team with access to legal search engines can get, and we have provided the full coding scheme for the laws so that other research teams could themselves replicate, add to and extend the score. No other score has done that, and thus other research teams cannot fully replicate their findings. While we do not contest that experts in the alcohol policy field add to the assessment of individual policies, we believe our method provides researchers with a new, more easily replicable tool to assess the changing landscape of state-level alcohol policies.

Our findings highlight differences in how population subgroups respond to the strength of the alcohol policy environment. As we show, men in every race/ethnic subgroup have higher probabilities of any binge drinking than women, although the gender gap narrows substantively in states with stronger policies, which could reflect state-level differences in the prevalence of these behaviors or as a result of the policies themselves.

Our findings confirm the association of individual and state-level factors to binge drinking outcomes, irrespective of the policy environment. In models estimating the prevalence of binge drinking for the population overall, the magnitude of the odds for gender, age, race/ethnicity, employment status and income were far greater than that of the state alcohol policy environment. Models that examine only current drinkers could reveal different patterns.

Finally, we have demonstrated that the use of a composite score such as the SAPS can be helpful in quantifying the entirety of policy-related approached that states use to reduce alcohol consumption and in identifying gaps across different states. While much of the current evidence base is focused on assessing the impact of new alcohol policies, such studies may fail to control for potential synergistic (or antagonistic) relationships among existing alcohol policies which, in turn, could lead to over- or under-specification of policy impacts.

### Limitations

This study has several important limitations. First, none of the three scores fully captures the entire picture of how effective a state is in regulating harmful alcohol consumption for its population. For instance, measures of policy enforcement are difficult to assess [23], as is the degree to which policies adopted capture the specific social norms that put its population at risk. Second, neither score includes pre-emption laws that curtail the ability of localities to make policies regarding the licensing and zoning of alcohol retailers. Third, because the SAPS is an index, the score may represent different policy packages in different states that add to the same value. However, this is a limitation of Naimi’s policy score as well, and the ability of our score to replicate their findings of these suggests that the scores are comparable. Fourth, our results represent the overall impact of the alcohol policy environment and do not provide estimates of the impacts of any single policy.

While our findings largely replicate those of Naimi et al [8] and Xuan et al. [9], the latter uses different methods of estimating multivariable models (GEE). While our analysis controlled for state-level population density, Xuan et al. used a measure of the proportion of urban population in each state. We did not control for the religious composition of each state, which could explain differences in our sub-group estimates for individuals ages 18–20 or for Hispanics. However, if the differences in these two covariates between our models were substantively meaningful, we would not expect to have substantially reproduced Xuan et al.’s findings.

Finally, we note that both scores do not fully capture policy implementation and enforcement at the city and county levels since accessible and frequently collected measures of these constructs at both state and local levels do not currently exist.

## Conclusion

State policymakers and advocates require up-to-date evidence on alcohol laws regarding their prevalence and overall effectiveness. Creating tools such as the SAPS which are replicable, extendable, and transparent can assist stakeholders to understand the implications and contributions of new laws to reducing excessive and harmful alcohol consumption. Persuading states with few or only weak alcohol policies to adopt the full range of evidence-based approaches can significantly contribute to reducing alcohol-related harms.

## Supporting information

S1 Table Components and scoring of individual alcohol-related laws and construction of the State Alcohol Policy Score (SAPS) (coding scheme). ^a^Types of exemptions for hours bans include geographical exemptions (e.g. certain county can choose to opt out or have more lenient hours), special day exemptions (e.g. sales allowed on Sunday before Christmas), exemptions by types of alcohol (e.g. more lenient hours for beer/wine), and exemptions by venue or vendor (e.g. exemptions for banquet, cabaret, or caterer).
^b^For dram shop law, the attorney searched for any law that allowed criminal, administrative or civil remedies against alcohol vendors for providing alcohol to intoxicated persons during the study period. While the binary 0/1 coding was suitable for criminal/administrative sanctions, availability of civil remedies was more difficult to determine due to the possibility of litigating under common law even in the absence of supporting statutes. As a result, the availability of civil remedies was coded in the following manner: 0 = if civil action is explicitly forbidden by statute; 0.5 = civil action may be allowed under common law; 1 = while technically allowed, the availability of civil action is by statute limited to certain cases; and 2 = civil action is explicitly allowed and supported by statute. In addition, both civil and criminal statutes often had varying degrees of intent or knowledge of wrongdoing for vendors to be held liable, which may greatly affect the chance of successful sanction and/or litigation.
^c^Among varying types of DUI penalties, the penalties with the strongest severity for the first-time offenders were selected. Empirical evidence supporting the positive effect of those sanctions on reducing DUI was more readily available across states than alternative or intermediate sanctions such as counseling and community service. The following penalties were included in our study: prison or jail; fine; license suspension; and vehicle seizure or impoundment. Based on the review of the primary law text, each of these DUI penalties was coded into two variables: (1) whether the penalty existed for the first-time offender in a given year (which was given a numerical value of 2 if the penalty is mandated (without the possibility for a judge’s discretion to not impose the penalty), 1 if the penalty existed but could be substituted by other penalty, 0 if no such penalty exists); and (2) the mandatory minimum duration/amount of penalty required by law (number of days or dollar amount).
^d^The law mandating ignition interlock installment was, while technically a part of the DUI penalties, included as a separate independent category in the score due to the complexity of the law. The mandatory interlock requirement often only applied to a certain subset of DUI offenders. As a result, in order to accurately reflect the scope of the law in each state, the research team examined the availability of the law separately for the following groups: (1) first time offenders; (2) repeat offenders; (3) aggravated offenders (e.g. an offender with a particularly high BAC level). For each group, the interlock variable was given a numerical value of 2 if mandatory law existed,1 if the law only existed as a substitution for another type of penalty (such as prison), and 0 if no law existed or the law only existed as a discretionary option for the judge. The research team coded the mandatory minimum duration that would apply to any offenders subject to the ignition interlock requirement.
(DOCX)

S2 Table Comparison of state score quintiles, 2009. Note: 1 = lowest quintile (worst); 5 = highest quintile (best)
Green = same rank or differs by only 1 quintile
Yellow = differs by 2 quintiles
Red = differs by 3 quintiles.
(DOCX)

S3 Table Adjusted odds ratios (aORs) of binge drinking associated with a 10-percentage point increase in 1-year lagged SAPS, 2005–2010 (full model). Behavioral Risk Factor Surveillance System 2004–2009.
(DOCX)

S1 Fig Histogram Comparing SAPS, Erickson and Naimi policy scores, 2009. (TIFF)

S1 Data Supplemental Datafile (excel format): State alcohol policy scores by state 2004–2009. (XLSX)
