# Supplementary material for: Evaluating the relationship between binge drinking rates and a replicable measure of U.S. state alcohol policy environments
Source: PLoS One. 2019 Jun 25;14(6):e0218718. doi: 10.1371/journal.pone.0218718 (PMC6592603; doi:10.1371/journal.pone.0218718)
Supplement: S1 Table — aTypes of exemptions for hours bans include geographical exemptions (e.g. certain county can choose to opt out or have more lenient hours), special day exemptions (e.g. sales allowed on Sunday before Christmas), exemptions by types of alcohol (e.g. more lenient hours for beer/wine), and exemptions by venue or vendor (e.g. exemptions for banquet, cabaret, or caterer). bFor dram shop law, the attorney searched for any law that allowed criminal, administrative or civil remedies against alcohol vendors for providing alcohol to intoxicated persons during the study period. While the binary 0/1 coding was suitable for criminal/administrative sanctions, availability of civil remedies was more difficult to determine due to the possibility of litigating under common law even in the absence of supporting statutes. As a result, the availability of civil remedies was coded in the following manner: 0 = if civil action is explicitly forbidden by statute; 0.5 = civil action may be allowed under common law; 1 = while technically allowed, the availability of civil action is by statute limited to certain cases; and 2 = civil action is explicitly allowed and supported by statute. In addition, both civil and criminal statutes often had varying degrees of intent or knowledge of wrongdoing for vendors to be held liable, which may greatly affect the chance of successful sanction and/or litigation. cAmong varying types of DUI penalties, the penalties with the strongest severity for the first-time offenders were selected. Empirical evidence supporting the positive effect of those sanctions on reducing DUI was more readily available across states than alternative or intermediate sanctions such as counseling and community service. The following penalties were included in our study: prison or jail; fine; license suspension; and vehicle seizure or impoundment. Based on the review of the primary law text, each of these DUI penalties was coded into two variables: (1) whether the penalty existed fo [file pone.0218718.s001.docx]

**S1 Table. Components and scoring of individual alcohol-related laws and construction of the State Alcohol Policy Score (SAPS) (coding scheme)**

| **Law** | **Component** | **Component description and point break down** | **Total available points** | **Mean score, 2009 (sd)**  **[Min, max]** |
| --- | --- | --- | --- | --- |
| **Alcohol pricing sub-score** | | | 31 | **24.79 (11.18)**  **[5.6, 53.57]** |
| Alcohol control systems  (source = APIS) | Beer control system  (available pts = 2) | State controls prices and profits of beer sales = 2 pts  Mixture of state controlled vendors and licensed private vendors for the sale of beer = 1 pt  State issues beer sale licenses to private vendors = 0 pts | 6 |  |
|  | Wine control system  (available pts = 2) | State controls prices and profits of wine sales = 2 points  Mixture of state controlled vendors and licensed private vendors for the sale of wine = 1 pt  State issues wine sale licenses to private vendors = 0 points |  |  |
|  | Spirits control system  (available pts = 2) | State controls prices and profits of spirits sales = 2 points  Mixture of state controlled vendors and licensed private vendors for the sale of spirits=1 pt  State issues spirit sale licenses to private vendors = 0 points |  |  |
| Total taxes (for beer)  (source = APIS, CCER COLI) | Total tax for beer | Using the average cost of a 6-pack of beer in each state-year, we calculated the total tax (sum of volume-based, ad valorem and sales taxes) for beer. We then rescaled the total tax to range from 0 to 1 within each year. | n/a |  |
| Wholesale pricing practices and restrictions  (source = APIS) | Beer volume discount laws  (available pts = 2) | State bans wholesalers from offering a discount to retailers based on volume/quantity of beer = 2 pts  State places limits on the quantity for which a discount may be offered for beer = 1 pt  No state law prohibits wholesalers from offering quantity discounts on beer = 0 pts | 18 |  |
|  | Wine volume discount laws  (available pts = 2) | State bans wholesalers from offering a discount to retailers based on volume/quantity of wine = 2 pts  State places limits on the quantity for which a discount may be offered for wine = 1 pt  No state law prohibits wholesalers from offering quantity discounts on wine = 0 pts |  |  |
|  | Spirits volume discount laws  (available pts = 2) | State bans wholesalers from offering a discount to retailers based on volume/quantity of spirits = 2 pts  State places limits on the quantity for which a discount may be offered for spirits = 1 pt  No state law prohibits wholesalers from offering quantity discounts on spirits = 0 pts |  |  |
|  | Beer post/hold laws  (available pts = 2) | State requires wholesalers to post beer prices and hold the prices for a specified period of time = 2 pts  State requires wholesalers to post beer prices, but a “hold” period is not stated/clearly defined = 1 pt  No state law requires the posting and holding of wholesale prices for a specified period of time = 0 pts |  |  |
|  | Wine post/hold laws  (available pts = 2) | State requires wholesalers to post wine prices and hold the prices for a specified period of time= 2 pts  State requires wholesalers to post wine prices, but a “hold” period is not stated/clearly defined = 1 pt  No state law requires the posting and holding of wholesale prices for a specified period of time = 0 pts |  |  |
|  | Spirits post/hold laws  (available pts = 2) | State requires wholesalers to post spirits prices and hold the prices for a specified period of time= 2 pts  State requires wholesalers to post spirits prices, but a “hold” period is not stated/clearly defined = 1 pt  No state law requires the posting and holding of wholesale prices for a specified period of time = 0 pts |  |  |
|  | Retailer credit beer  (available pts = 1) | State prohibits wholesalers from extending credit to retailers and requires that either wholesaler transactions must be in cash or that payment is due prior to or at the time of delivery = 1 pt  State allows wholesalers to extend credit to retailers for beer = 0 pts |  |  |
|  | Retailer credit wine  (available pts = 1) | State prohibits wholesalers from extending credit to retailers and requires that either wholesaler transactions must be in cash or that payment is due prior to or at the time of delivery = 1 pt  State allows wholesalers to extend credit to retailers for wine = 0 pts |  |  |
|  | Retailer credit spirits  (available pts = 1) | State prohibits wholesalers from extending credit to retailers and requires that either wholesaler transactions must be in cash or that payment is due prior to or at the time of delivery = 1 pt  State allows wholesalers to extend credit to retailers for spirits = 0 pts |  |  |
|  | Minimum markup - beer  (available pts = 1) | State does not allow wholesalers to sell beer below cost or requires that wholesalers establish a minimum markup or maximum discount for beer sold to retailers = 1 pt  No minimum markup restriction for beer = 0 pts |  |  |
|  | Minimum markup - wine  (available pts = 1) | State does not allow wholesalers to sell wine below cost or requires that wholesalers establish a minimum markup or maximum discount for wine sold to retailers = 1 pt  No minimum markup restriction for wine = 0 pts |  |  |
|  | Minimum markup - spirits  (available pts = 1) | State does not allow wholesalers to sell beer below cost or requires that wholesalers establish a minimum markup or maximum discount for beer sold to retailers = 1 pt  No minimum markup restriction for beer = 0 pts |  |  |
| Retail price restrictions  (source = APIS) | Happy hours – reduced price  (available pts = 2) | State bans offering a discount/price promotion to customers at any time during hours of operation = 2 pts  State restricts offering a discount/price promotion to customers at any time during hours of operation = 1 pt  No state law banning or restricting price promotions = 0 pts | 7 |  |
|  | Free beverages  (available pts = 1) | State bans providing customers with free beverages either as a promotion or case-by-case basis = 1 pt  No state law banning free beverages = 0 pts |  |  |
|  | Multiple servings for a single price  (available pts = 1) | State bans offering additional drinks for the same price as a single drink (e.g., two-for-ones) = 1 pt  No state law banning two-for-ones, etc. = 0 pts |  |  |
|  | Multiple servings at one time  (available pts = 1) | State bans service of more than one drink to a customer at a time = 1 pt  No state law banning serving multiple drinks = 0 pts |  |  |
|  | Unlimited beverages for a fixed price or period  (available pts = 1) | State bans instituting a fixed price for an unlimited amount of drinks during a fixed period of time (e.g., all-you-can-drink) = 1 pt  No state law banning all-you-can-drink, etc. = 0 pts |  |  |
|  | Increased volume without increase in price  (available pts = 1) | State bans offering drinks with increased amounts of alcohol at the same price as regular-sized drinks (e.g., double shots for the price of single shots) = 1 pt  No state law banning double shots, etc. = 0 pts |  |  |
| **Sales/retailer restrictions sub-score** | | | 36 | **36.04 (13.43)**  **[10.71,73.66]** |
| Sunday sales ban  (source = APIS) | Sunday sales ban  (available pts = 2) | State completely bans Sunday sales of alcoholic beverages for off-premises consumption = 2 pts  State has law banning Sunday sales of alcoholic beverages for off-premises consumption with exceptions = 1 pt  No state law banning Sunday sales = 0 pts | 2 |  |
| The number of hours during which alcohol cannot be sold  (original legal research) | Hours ban for on premise consumption  (available pts = 2) | The total number of hours/week that a state prohibits vendors from selling alcohol for on premise consumption is above the median = 2 pts  The total number of hours/week that a state prohibits vendors from selling alcohol for on premise consumption is below the median = 1 pt  No state law limiting number of hours/week vendors can sell alcohol for on premise consumption = 0 pts | 7 |  |
|  | Hours ban for off premise consumption  (available pts = 2) | The total number of hours/week that a state prohibits vendors from selling alcohol for off premise consumption is above the median = 2 pts  The total number of hours/week that a state prohibits vendors from selling alcohol for off premise consumption is below the median = 1 pt  No state law limiting number of hours/week vendors can sell alcohol for off premise consumption = 0 pts |  |  |
|  | Hours ban exemption^a^  (available pts = 3) | State does not allow any exemptions to hours ban = 3 pts  State allows 1 exemptions to hours ban = 2 pts  State allows 2 exemptions to hours ban = 1 pts  State allows 3 or more exemptions to hours ban = 0 pts |  |  |
| Keg registration  (source = APIS) | Total prohibition on keg sales  (available pts = 4) | State has full law that completely prohibits the purchase and sale of kegs = 4 pts  No state law prohibiting keg sales = 0 pts | 4 |  |
|  | Unregistered kegs  (available pts = 1) | State prohibits possession of an unregistered or unlabeled keg = 1 pt  No state law banning possession of an unregistered or unlabeled keg = 0 pts |  |  |
|  | Keg label  (available pts = 1) | State prohibits the destruction of the label on a keg = 1 pt  No state law banning keg label destruction = 0 pts. |  |  |
|  | Address where keg was consumed  (available pts = 1) | State requires an address where keg will be consumed = 1 pt  No state law requiring the address = 0 pts |  |  |
| Beverage service training  (source = APIS) | Require beverage service training  (available pts = 4) | State law specifies that beverage service training is mandatory for everyone = 4 pts  State law specifies that beverage service training is mandatory but not for everyone = 3 pts  State law specifies that beverage service training is voluntary, incentives provided = 2 pts  State law specifies that beverage service training is voluntary, no incentives provided=1 pt  No state law requiring mandatory or voluntary beverage service training = 0 pts | 8 |  |
|  | On/off premise establishments  (available pts = 2) | State beverage service training law applies to both on-premises establishments and off-premises establishments = 2 pts  State beverage service training law applies to either on-premises or off-premises establishments, but not both, or is unspecified = 1 pt  No state law regarding on/off premise beverage service training = 0 pts |  |  |
|  | New/existing licensees  (available pts = 2) | State beverage training law applies to both new and existing licensees = 2 pts  State beverage training law applies to either new or existing licensees, not both, or is unspecified = 1 pt  No state law regarding new/existing licensees = 0 pts |  |  |
| Dram shop laws^b^  (original legal research) | Dram shop liability  (available pts = 1) | State law allows alcohol vendors to be criminally, administratively or civil action-wise responsible for providing alcohol to intoxicated person = 1 point  Civil liability may exist under common law but no statute on the issue = 0.5 pts  No dram shop liability law = 0 pts | 12 |  |
|  | Criminal sanctions  (available pts = 1) | State law allows for criminal or administrative penalty for vendors who provide alcohol to intoxicated persons = 1 pt  State law does not allow criminal or administrative sanctions = 0 pts |  |  |
|  | Civil remedy  (available pts = 2) | State law supports and explicitly allows civil action against vendors who provide alcohol to intoxicated persons = 2 pts  State law allows civil action against vendors who provide alcohol to intoxicated persons, but it is explicitly limited = 1 pt  Civil action against vendors may be allowed under common law = 0.5 pts  State law does not allow civil remedies = 0 pts |  |  |
|  | Criminal scienter  (available pts = 4) | No scienter required by state law. If the person was intoxicated and/or if serving of alcohol led to intoxication, vendor is liable = 4 pts  Reasonable person standard under state law. Irrespective of what the vendor’s actual intent was, they are held liable if a reasonable person would have found the person intoxicated under the same circumstances = 3 pts  Implied/explicit knowledge of intoxication required under state law. The server/vender knew or should have known about intoxication, or the person must have been "clearly" "visibly" "obviously" intoxicated = 2 pts  Explicit knowledge of intoxication or intent required under state law. The server/vendor must have had “actual knowledge" of intoxication, criminal negligence, “willfully and knowingly” served alcohol, or had knowledge that the person will drive him/herself home, etc. = 1 pt |  |  |
|  | Civil tort law intent  (available pts = 4) | No intent required to find vendor liable under state law. If the person was intoxicated and/or if serving of alcohol led to intoxication, vendor is liable = 4 pts  Reasonable person standard in state law. Irrespective of what the vendor’s actual intent was, they are held liable if a reasonable person would have found the person intoxicated under the same circumstances = 3 pts  Implied/explicit knowledge of intoxication required by state law. The server/vender knew or should have known about intoxication, or the person must have been "clearly" "visibly" "obviously" intoxicated = 2 pts  Explicit knowledge of intoxication or intent required under state law. The server/vendor must have had “actual knowledge" of intoxication, criminal negligence, “willfully and knowingly” served alcohol, or had knowledge that the person will drive him/herself home, etc. = 1 pt |  |  |
| Pregnancy and alcohol warning signs  (source = APIS) | Fetal alcohol syndrome warning signs  (available pts = 3) | State law requires warning signs at both on-sale and off-sale establishments = 3 pts  State law requires warning signs at off-sale establishments only = 2 pts  State law requires warning signs at on-sale establishments only = 1 pt  No state law regarding on-sale nor off-sale warning signs= 0 pts | 3 |  |
| **Driving sub-score** | | |  | **58.49 (11.74)**  **[23.27, 82.57]** |
| Open container laws  (source = APIS) | Open containers of alcoholic beverages prohibited in the passenger compartments of non-commercial motor vehicle  (available pts = 2) | State prohibition of open containers applies to all passenger areas of any motor vehicles, all alcoholic beverages, all occupants, and any public highway or right of way = 2 pts  State prohibition does not apply to all five of the previously listed factors = 1 pt  No state law prohibiting open containers = 0 pts | 3 |  |
|  | Enforcement of open container laws  (available pts = 1) | State gives law enforcement officers the authority to enforce the law without probable cause = 1 pt  No state law allowing primary enforcement = 0 pts |  |  |
| DUI penalties^c^  (original legal research) | Imprisonment first offense  (available pts = 2) | State law requires imprisonment as part of mandatory penalty for first DUI offense = 2 pts  State law stipulates that prison or other penalty is mandatory (judge can choose one or the other) or, prison can be substituted by other penalty (in part or whole) = 1 pt  No or discretionary state law regarding imprisonment for first offense DUI = 0 pts | 11 |  |
|  | License suspension first offense  (available pts = 2) | State law requires license suspension to be part of the mandatory penalty for first DUI offense = 2 pts  State law stipulates that license revocation or other penalty is mandatory (judge can choose) or, while mandatory, license revocation can be substituted by other penalty (in part or whole) = 1 pt  No or discretionary state law regarding license suspension for first offense DUI = 0 pts |  |  |
|  | Days of license suspension  (available pts = 2) | Minimum number of days the license of the first-time DUI offender must be suspended > 90 (based on the penalty license revocation rather than administrative license revocation) = 2 pts  Minimum number of days the license of the first-time DUI offender must be suspended ≤ 90 (based on the penalty license revocation rather than administrative license revocation) = 1 pt  No minimum number of days the license of the first-time DUI offender must be suspended (based on the penalty license revocation rather than administrative license revocation =0 pts |  |  |
|  | Fine first offense  (available pts = 2) | State law requires fine to be part of the mandatory penalty for first DUI offense = 2 pts  State law stipulates that fine or other penalty is mandatory (judge can choose one or the other) or, while mandatory, fine can be substituted by other penalty (in part or whole)= 1 pt  No or discretionary state law regarding fine for first offense DUI = 0 pts |  |  |
|  | Vehicle seizure first offense  (available pts = 2) | State law requires vehicle seizure or impoundment to be part of the mandatory penalty for first DUI offense = 2 pts  State law stipulates that vehicle seizure or impoundment or other penalty is mandatory (judge can choose one or the other) or, while mandatory, vehicle seizure/impoundment can be substituted by other penalty (in part or whole)= 1 pt  No or discretionary state law regarding vehicle seizure or impoundment for first DUI offense = 0 pts |  |  |
|  | Sobriety check points  (available pts = 1) | State law allows for sobriety check points = 1 pt  State law does not allow for sobriety check points = 0 pts |  |  |
| Ignition interlock installation requirement^d^  (source = original legal research) | Ignition lock for aggravated DUI  (available pts = 2) | State requires mandatory ignition interlock after the license suspension period for aggravated DUI offenders = 2 pts  Ignition interlock exists as substitution of other penalty (often license revocation) for aggravated DUI offenders = 1 pt  No state interlock law or law is discretionary for aggravated DUI offenders = 0 pts | 7 |  |
|  | Ignition lock for first time DUI  (available pts = 2) | State requires mandatory ignition interlock after the license suspension period for first time DUI offenders = 2 pts  Ignition interlock exists as substitution of other penalty (often license revocation) for first time DUI offenders = 1 pt  No state interlock law or law is discretionary for first time DUI offenders = 0 pts |  |  |
|  | Ignition lock for repeat DUI  (available pts = 2) | State requires mandatory ignition interlock after the license suspension period for repeat DUI offenders = 2 pts  Ignition interlock exists as substitution of other penalty (often license revocation) for repeat DUI offenders = 1 pt  No state interlock law or law is discretionary for repeat DUI offenders = 0 pts |  |  |
|  | Minimum number of days for ignition lock  (available pts = 1) | State law requires a minimum number of days that first time DUI offenders must use ignition interlock when they drive = 1 pt  No state requirement for minimum number of days that first time DUI offenders must use ignition interlock when they drive = 0 pts |  |  |
| Graduated Driving Laws  (Source: IIHS) | GDL permit age  (available pts =1) | GDL permit age is 16 = 1 pt  GDL permit age is less than 16 = 0 pts |  |  |
|  | GDL permit holding days  (available pts = 2) | Drivers must hold a learning permit for at minimum of 6 months to be eligible for a license = 2 pts  Drivers must hold a learning permit for less than 6 months to be eligible for a license = 1 pt  No holding days required = 0 pts |  |  |
|  | GDL total practice hours  (available pts = 2) | Drivers must complete a minimum of 50 certified driving practice hours before eligible for a license = 2 pts  Drivers must complete less than 50 certified driving practice hours before eligible for a license = 1 pt  No minimum practice hours required = 0 pts | 13 |  |
|  | GDL nighttime practice hours  (available pts = 1) | State law stipulates that a minimum of 10 practice hours must be completed at night = 1 pt  State law stipulates that less 10 practice hours must be completed at night or there is no nighttime practice requirement = 0 pt |  |  |
|  | GDL nighttime driving ban  (available pts =4) | Drivers in intermediate stage have a nighttime driving ban that begins by 8pm = 4 pts  Drivers in intermediate stage have a nighttime driving ban between 8:01pm and 10pm = 3 pts  Drivers in intermediate stage have a nighttime driving ban between 10:01pm and 12am = 2 pts  Drivers in intermediate stage have a nighttime driving ban after 12am = 1 pt  No nighttime driving ban for drivers in intermediate stage = 0 pts |  |  |
|  | GDL nighttime ban lift age  (available pts = 2) | Nighttime driving ban lift age is 18 = 2 pts  Nighttime driving ban lift age is 17 = 1pts  Nighttime driving ban life age if less than 17 or there is no nighttime driving ban = 0 pts |  |  |
|  | GDL teen passenger ban  (available pts = 1) | State law prohibits teen passengers during intermediate stage = 1 pt  No restriction on teen passengers = 0 pts |  |  |
| **Underage (<21) sub-score** | | | | **47.88 (13.91)**  **[24.81, 94.48]** |
| Providing alcohol to minors (source= APIS) | Furnishing alcohol to minors  (available pts = 4) | State law prohibits all furnishing of alcohol to minors = 4 pts  State law allows an exemption if alcohol is furnished by parent/guardian only in own home, private residence, or private location = 3 pts  State law allows an exemption if alcohol is furnished by parent/guardian without regard to location = 2 pts  State law allows affirmative defense (Facts and arguments that exonerate a defendant, even if all allegations in the complaint are true) = 1 pt |  |  |
|  | Host liability action type  (available pts = 3) | State law holds hosts responsible if underage guests possess alcohol = 3 pts  State law holds hosts responsible if underage guests consume alcohol = 2 pts  State law holds hosts responsible if underage guests consume alcohol and are intoxicated= 1 pt  No state law imposes responsibility on social hosts = 0 pts |  |  |
|  | Host liability exemption type  (available pts = 2) | State law does not allow any exemptions for family members and/or non-owner residents of the property = 2 pts  State law provides exemption to either family member (or non-family member) or non-owners = 1 pt  No state law imposes responsibility on social hosts = 0 pts |  |  |
|  | Host liability party types  (available pts = 2) | State law applies to all party types = 2pts  State law applied to underage parties only = 1 pt  No state law imposes responsibility on social hosts = 0 pts |  |  |
|  | Host liability residence type  (available pts = 3) | State law applies to residential property, outdoor property and other types of property = 3 pts  State law applies to 2 of these types of properties = 2 pts  State law only applies to only 1 of these types of properties = 1 pt  No state law imposes responsibility on social hosts = 0 pts |  |  |
| Server laws (source- APIS) | Minimum age to sell beer and wine, off premises  (available pts = 2) | Minimum age to sell beer and wine off premises is 21 = 2 pts  Minimum age to sell beer and wine off premises is under 21 but manager must be present = 1 pt  Minimum age to sell beer and wine off premises is under 21, no manager requirement = 0 pts |  |  |
|  | Minimum age to sells spirits, off premises  (available pts = 2) | Minimum age to sell spirits off premises is 21 = 2 pts  Minimum age to sell spirits off premises is under 21 but manager must be present = 1 pt  Minimum age to sell spirits off premises is under 21, no manager requirement = 0 pts |  |  |
|  | Minimum age to serve beer, wine, and spirits, on premises  (available pts = 2) | Minimum age to serve beer, wine and spirits on premises is 21 = 2 pts  Minimum age to serve beer, wine and spirits on premises is under 21 but manager must be present = 1 pt  Minimum age to serve beer, wine and spirits on premises is under 21, no manager requirement = 0 pts |  |  |
|  | Minimum age to bartend beer, wine, and spirits, on premises  (available pts = 2) | Minimum age to bartend beer, wine and spirits on premises is 21 = 2 pts  Minimum age to bartend beer, wine and spirits on premises is under 21 but manager must be present = 1 pt  Minimum age to bartend beer, wine and spirits on premises is under 21, no manager requirement = 0 pts |  |  |
| Underage (<21) identification laws | Criminal offense to use false ID  (available pts = 1) | State law makes it a criminal offense to use a false ID to purchase alcohol = 1 pt  No state law making it a criminal offense = 0 pts |  |  |
|  | License suspension for using false ID  (available pts = 3) | State law imposes an administrative and judicial license suspension for using a false ID = 3 pts  State law imposes an administrative license suspension for using a false ID = 2 pts  State law imposes a judicial license suspension for using a false ID = 1 pt  No license suspension laws regarding the use of false ID = 0 pts |  |  |
|  | Lend/transfer/sell false ID  (available pts = 1) | State law makes it a criminal offense to lend/transfer/sell a false ID = 1 pt  No state law regarding lend/transfer/sell a false ID = 0 pts |  |  |
|  | Alter/manufacture false ID  (available pts = 1) | State law makes it criminal offense to alter or manufacture a false ID = 1 pt  No state law regarding altering or manufacturing false ID = 0 pts |  |  |
|  | Electronic scanners  (available pts = 1) | State law provides incentives for use of electronic scanners to identify false IDs = 1 pt  No state law providing incentives = 0 pts |  |  |
|  | Distinctive licenses  (available pts = 1) | State law stipulates that driver's licenses for persons under 21 must be easily distinguishable from licenses from persons older than 21 = 1 pt  No state law regarding distinctive licenses = 0 pts |  |  |
|  | False ID seizure  (available pts = 1) | State law allows retailers to seize apparently false IDs = 1 pt  No state law allowing false ID seizure by retailers = 0 pts |  |  |
|  | Affirmative defense  (available pts = 3) | State laws does not allow affirmative defense for retailers = 3 pts  State law allows specific affirmative defense, meaning that the retailer inspected false ID and concluded it was valid = 2 pts  State law allows general affirmative defense, meaning that the retailer concluded purchaser was above 21 = 1 pt |  |  |
|  | Right to sue  (available pts = 1) | State law provides retailer right to sue the minor who used false ID = 1 pt  No state law allowing right to sue = 0 pts |  |  |
|  | Detain minor  (available pts = 1) | State law provides retailer right to detain the minor who used false ID=1 pt  No state law allowing right to detain = 0 pts |  |  |
|  | Purchase leads to license suspension/revocation  (available pts = 1) | State law stipulates that purchase of alcohol leads to license suspension or revocation – 1 pt  No state law regarding license suspension/revocation for purchasing = 0 pts |  |  |
|  | Possession leads to license suspension/revocation  (available pts = 1) | State law stipulates that possession of alcohol leads to license suspension or revocation – 1 pt  No state law regarding license suspension/revocation for possession = 0 pts |  |  |
|  | Consumption leads to license suspension/revocation  (available pts = 1) | State law stipulates that consumption of alcohol leads to license suspension or revocation – 1 pt  No state law regarding license suspension/revocation for consumption = 0 pts |  |  |
|  | Authority to sanction  (available pts = 3) | State law allows license sanctions to be mandatory = 3 pt  Authority to impose license sanctions is mandatory for some ages but discretionary for other ages = 2 pts  Authority to impose license sanctions discretionary for all ages= 1 pt  No state laws regarding authority to sanction = 0 pts |  |  |
|  | Age limit for sanctions  (available pts =3) | Age limit is 21 for all sanctions =3 pts  Age limit is 21 for some sanctions and less than 21 for other sanctions ==2 pts  Age limit is less than 21 for all sanctions ==3 pts  No state law regarding sanctions =0 pts |  |  |
|  | Minimum length of suspension/revocation of license  (available pts =2) | Minimum length of license suspension/revocation is greater than 90 days = 2 pts  Minimum length of license suspension/revocation is less than or equal to 90 days = 1 pt  No state law regarding minimum length = 0 pts |  |  |
| Underage (<21)  Possession laws (Source: APIS) | Underage (<21) consumption  (available pts = 4) | State law prohibits underage alcohol consumption= 4 pts  State law allows an exemption for minors if parent/guardian/spouse is present in own home, private residence, or private location = 3 pts  State law allows an exemption for minors if parent/guardian/spouse is present without regard to location = 2 pts  State law allows an exemption for specific locations = 1 pts  No state law prohibiting underage alcohol consumption = 0 pts |  |  |
|  | Underage (<21) possession  (available pts = 4) | State law prohibits underage alcohol possession = 4 pts  State law allows an exemption for minors if parent/guardian/spouse is present in own home, private residence, or private location = 3 pts  State law allows an exemption for minors if parent/guardian/spouse is present without regard to location = 2 pts  State law allows an exemption for specific locations = 1 pts  No state law prohibiting underage alcohol possession = 0 pts |  |  |
|  | Underage (<21) internal possession  (available pts = 4) | State law prohibits underage internal alcohol possession = 4 pts  State law allows an exemption for minors if parent/guardian/spouse is present in own home, private residence, or private location = 3 pts  State law allows an exemption for minors if parent/guardian/spouse is present without regard to location = 2 pts  State law allows an exemption for specific locations = 1 pts  No state law prohibiting underage internal alcohol possession = 0 pts |  |  |

^a^Types of exemptions for hours bans include geographical exemptions (e.g. certain county can choose to opt out or have more lenient hours), special day exemptions (e.g. sales allowed on Sunday before Christmas), exemptions by types of alcohol (e.g. more lenient hours for beer/wine), and exemptions by venue or vendor (e.g. exemptions for banquet, cabaret, or caterer).

^b^For dram shop law, the attorney searched for any law that allowed criminal, administrative or civil remedies against alcohol vendors for providing alcohol to intoxicated persons during the study period. While the binary 0/1 coding was suitable for criminal/administrative sanctions, availability of civil remedies was more difficult to determine due to the possibility of litigating under common law even in the absence of supporting statutes. As a result, the availability of civil remedies was coded in the following manner: 0= if civil action is explicitly forbidden by statute; 0.5= civil action may be allowed under common law; 1= while technically allowed, the availability of civil action is by statute limited to certain cases; and 2= civil action is explicitly allowed and supported by statute. In addition, both civil and criminal statutes often had varying degrees of intent or knowledge of wrongdoing for vendors to be held liable, which may greatly affect the chance of successful sanction and/or litigation.

^c^Among varying types of DUI penalties, the penalties with the strongest severity for the first-time offenders were selected. Empirical evidence supporting the positive effect of those sanctions on reducing DUI was more readily available across states than alternative or intermediate sanctions such as counseling and community service. The following penalties were included in our study: prison or jail; fine; license suspension; and vehicle seizure or impoundment. Based on the review of the primary law text, each of these DUI penalties was coded into two variables: (1) whether the penalty existed for the first-time offender in a given year (which was given a numerical value of 2 if the penalty is mandated (without the possibility for a judge’s discretion to not impose the penalty), 1 if the penalty existed but could be substituted by other penalty, 0 if no such penalty exists); and (2) the mandatory minimum duration/amount of penalty required by law (number of days or dollar amount).

^d^The law mandating ignition interlock installment was, while technically a part of the DUI penalties, included as a separate independent category in the score due to the complexity of the law. The mandatory interlock requirement often only applied to a certain subset of DUI offenders. As a result, in order to accurately reflect the scope of the law in each state, the research team examined the availability of the law separately for the following groups: (1) first time offenders; (2) repeat offenders; (3) aggravated offenders (e.g. an offender with a particularly high BAC level). For each group, the interlock variable was given a numerical value of 2 if mandatory law existed,1 if the law only existed as a substitution for another type of penalty (such as prison), and 0 if no law existed or the law only existed as a discretionary option for the judge. The research team coded the mandatory minimum duration that would apply to any offenders subject to the ignition interlock requirement.
