# Supplementary material for: Evaluating the relationship between binge drinking rates and a replicable measure of U.S. state alcohol policy environments
Source: PLoS One. 2019 Jun 25;14(6):e0218718. doi: 10.1371/journal.pone.0218718 (PMC6592603; doi:10.1371/journal.pone.0218718)
Supplement: S2 Table — Note: 1 = lowest quintile (worst); 5 = highest quintile (best) Green = same rank or differs by only 1 quintile Yellow = differs by 2 quintiles Red = differs by 3 quintiles. (DOCX) [file pone.0218718.s002.docx]

**S2 Table: Comparison of state score quintiles, 2009**

| state | SAPS | Naimi | Erickson |
| --- | --- | --- | --- |
| Mississippi | 1 | 2 | 1 |
| Wyoming | 1 | 1 | 1 |
| North Dakota | 1 | 1 | 1 |
| Iowa | 1 | 1 | 1 |
| Hawaii | 1 | 3 | 1 |
| Wisconsin | 1 | 1 | 1 |
| Nevada | 1 | 1 | 1 |
| Louisiana | 1 | 2 | 1 |
| Texas | 1 | 2 | 1 |
| West Virginia | 1 | 3 | 1 |
| Montana | 2 | 1 | 2 |
| Arkansas | 2 | 4 | 2 |
| Rhode Island | 2 | 2 | 2 |
| Maine | 2 | 4 | 2 |
| Idaho | 2 | 3 | 2 |
| Maryland | 2 | 1 | 2 |
| Colorado | 2 | 2 | 2 |
| Florida | 2 | 1 | 2 |
| Vermont | 2 | 5 | 2 |
| Missouri | 2 | 1 | 2 |
| Virginia | 3 | 2 | 3 |
| South Dakota | 3 | 1 | 3 |
| Kentucky | 3 | 3 | 3 |
| Massachusetts | 3 | 4 | 3 |
| New Mexico | 3 | 5 | 3 |
| Georgia | 3 | 3 | 3 |
| Michigan | 3 | 4 | 3 |
| Nebraska | 3 | 2 | 3 |
| Ohio | 3 | 4 | 3 |
| New Jersey | 3 | 2 | 3 |
| California | 4 | 1 | 4 |
| Alabama | 4 | 5 | 4 |
| New York | 4 | 2 | 4 |
| Connecticut | 4 | 4 | 4 |
| South Carolina | 4 | 3 | 4 |
| Minnesota | 4 | 3 | 4 |
| Indiana | 4 | 3 | 4 |
| Oklahoma | 4 | 5 | 4 |
| Alaska | 4 | 2 | 4 |
| Arizona | 4 | 4 | 4 |
| Delaware | 5 | 3 | 5 |
| Pennsylvania | 5 | 5 | 5 |
| Tennessee | 5 | 5 | 5 |
| New Hampshire | 5 | 5 | 5 |
| Illinois | 5 | 3 | 5 |
| Kansas | 5 | 5 | 5 |
| North Carolina | 5 | 4 | 5 |
| Washington | 5 | 5 | 5 |
| Oregon | 5 | 4 | 5 |
| Utah | 5 | 5 | 5 |

Note: 1 = lowest quintile (worst); 5=highest quintile (best)

Green= same rank or differs by only 1 quintile

Yellow = differs by 2 quintiles

Red= differs by 3 quintiles
