# Supplementary material for: Evaluating the relationship between binge drinking rates and a replicable measure of U.S. state alcohol policy environments
Source: PLoS One. 2019 Jun 25;14(6):e0218718. doi: 10.1371/journal.pone.0218718 (PMC6592603; doi:10.1371/journal.pone.0218718)
Supplement: S3 Table — Behavioral Risk Factor Surveillance System 2004–2009. (DOCX) [file pone.0218718.s003.docx]

**S3 Table: Adjusted odds ratios (aORs) of binge drinking associated with a 10-percentage point increase in 1-year lagged SAPS, 2005-2010 (full model)**

|  | aOR | P>t | [95% Conf. | Interval] |
| --- | --- | --- | --- | --- |
| SAPS score | 0.911 | 0.000 | 0.898 | 0.924 |
| Male | 2.346 | 0.000 | 2.302 | 2.391 |
| High school graduate (v less) | 0.981 | 0.408 | 0.938 | 1.026 |
| Some college | 0.927 | 0.001 | 0.886 | 0.970 |
| College graduate | 0.741 | 0.000 | 0.708 | 0.776 |
| Age 21-34 (v 18-20) | 1.998 | 0.000 | 1.867 | 2.137 |
| Age 35-64 | 1.008 | 0.804 | 0.943 | 1.078 |
| Age 65+ | 0.327 | 0.000 | 0.304 | 0.353 |
| NH black (v NH white) | 0.569 | 0.000 | 0.546 | 0.592 |
| NH Other races | 0.606 | 0.000 | 0.578 | 0.636 |
| Hispanic | 0.867 | 0.000 | 0.833 | 0.902 |
| Married/partnered | 0.638 | 0.000 | 0.624 | 0.653 |
| HH income 25-49.9k (v <25k) | 1.200 | 0.000 | 1.161 | 1.239 |
| 50,000 - 74,999 | 1.338 | 0.000 | 1.292 | 1.387 |
| 75,000+ | 1.642 | 0.000 | 1.585 | 1.702 |
| Self-employed | 0.965 | 0.026 | 0.935 | 0.996 |
| Out of work | 1.037 | 0.100 | 0.993 | 1.083 |
| Homemaker | 0.591 | 0.000 | 0.565 | 0.618 |
| Student | 0.950 | 0.107 | 0.893 | 1.011 |
| Retired | 0.639 | 0.000 | 0.617 | 0.662 |
| Unable to work | 0.450 | 0.000 | 0.426 | 0.477 |
| State/Percent poverty | 0.986 | 0.000 | 0.982 | 0.991 |
| State/police/1000 population | 0.998 | 0.838 | 0.983 | 1.014 |
| State/Log population size | 1.050 | 0.000 | 1.038 | 1.063 |
| State/Percent White | 0.995 | 0.000 | 0.994 | 0.996 |
| State/Percent 21 and over | 1.036 | 0.000 | 1.030 | 1.043 |
| State/Percent female | 0.872 | 0.000 | 0.849 | 0.896 |
| State/Population density | 1.000 | 0.000 | 1.000 | 1.000 |
| 2006 (v 2005) | 1.072 | 0.000 | 1.035 | 1.110 |
| 2007 | 1.142 | 0.000 | 1.104 | 1.180 |
| 2008 | 1.104 | 0.000 | 1.069 | 1.141 |
| 2009 | 1.141 | 0.000 | 1.103 | 1.179 |
| 2010 | 1.173 | 0.000 | 1.133 | 1.214 |
| Midwest region (v NE) | 1.031 | 0.099 | 0.994 | 1.070 |
| South | 0.743 | 0.000 | 0.716 | 0.770 |
| West | 0.743 | 0.000 | 0.705 | 0.784 |

Behavioral Risk Factor Surveillance System, 2004-2009
