# Supplementary figures and images for: Evaluating the relationship between binge drinking rates and a replicable measure of U.S. state alcohol policy environments
Source: PLoS One. 2019 Jun 25;14(6):e0218718. doi: 10.1371/journal.pone.0218718 (PMC6592603; doi:10.1371/journal.pone.0218718)

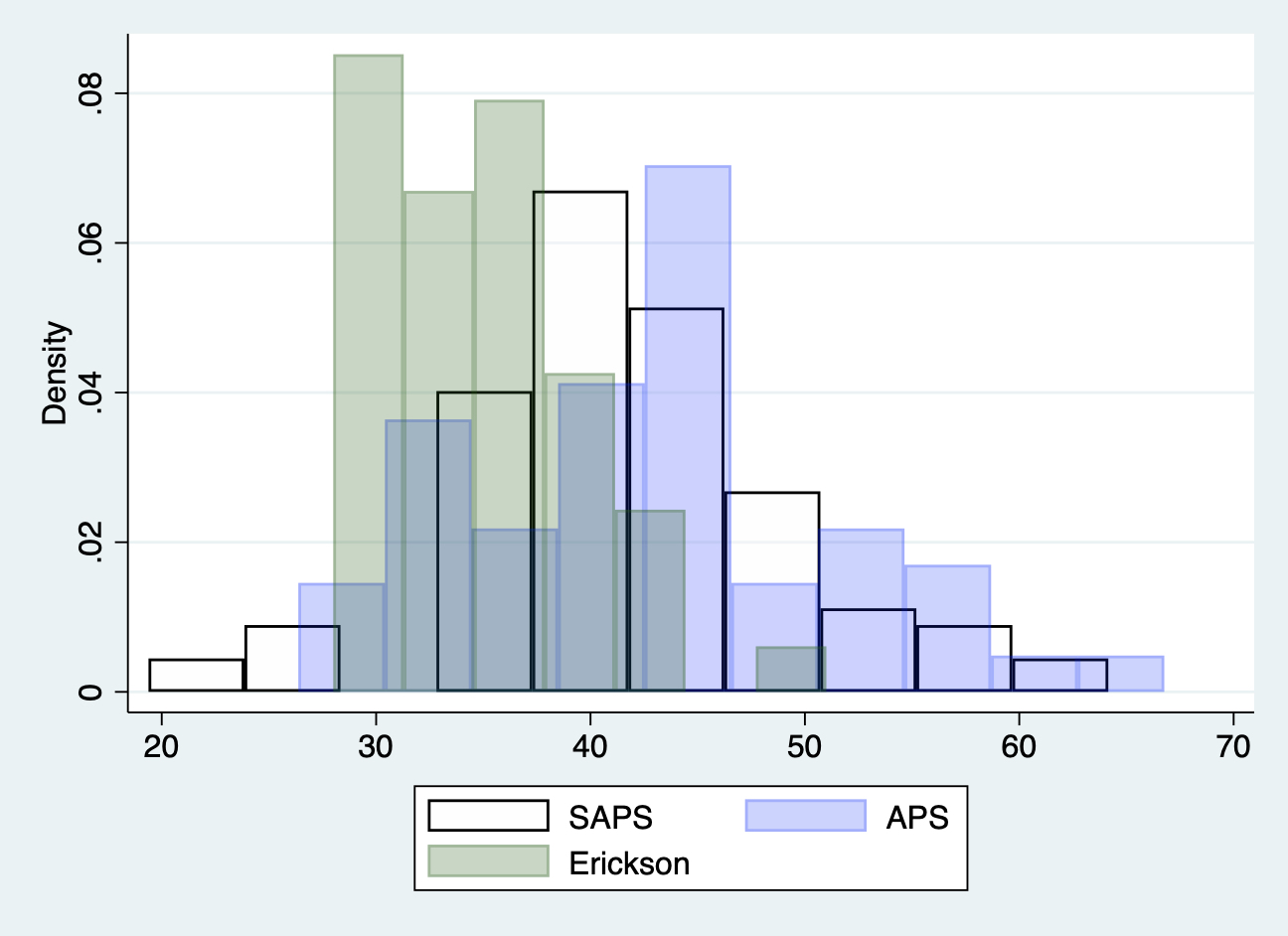

Supplement: S1 Fig — (TIFF) [file pone.0218718.s004.tiff]
